# 255 Structure, Staffing, and Strain: Lessons from U.S. Infection Preventionists on Building Resilient Systems Post–COVID-19

**DOI:** 10.1017/ash.2026.10623

**Published:** 2026-06-23

**Authors:** Lisa Casanova, Alexander Kirpich, Alicia Daubon, Nneka Odimgbe, Jesse Jacob

**Affiliations:** 1 Georgia State University; 2 Emory University

## Abstract

**Background:** Sinks in hospital patient rooms are potential reservoirs of hospital acquired infections (HAI) pathogens and outbreaks; sink colonization can occur in new buildings soon after patient occupancy and persist. To control this source, it is important to understand how occurrence and concentrations of organisms in sinks and drains change over time. This study longitudinally measures the occurrence and concentration of Acinetobacter, Pseudomonas aeruginosa, and Klebsiella pneumoniae in sinks and drains of new inpatient hospital rooms after patient occupancy. Methods Three inpatient units of a newly constructed cancer hospital were studied. Ten randomly selected rooms per unit were sampled biweekly for seven months for Acinetobacter, Pseudomonas aeruginosa, and Klebsiella pneumoniae. Surface samples were swabbed from a 4x4 cm section of the sink basin around the drain. Water was sampled from the p-trap via the drain. Results Approximately 400 sink and drain samples were taken. Acinetobacter was the most commonly detected organism, and Klebsiella the least. Based on the unit, 90-100% of sink and 50-90% of drain samples by the end of the study period had detectable Acinetobacter, 90-100% of sink and 50-80% of drain had Pseudomonas, and 0-10% of sink and 0-30% of drain had Klebsiella. In samples with detectable organisms, Acinetobacter concentrations ranged from 1.3-8 logCFU in sinks and 1.6-6.7 log CFU/mL in drains. Pseudomonas aeruginosa ranged from 2.8-7.4 log CFU in sinks and 2.2-6.1 log CFU/mL in drains, and Klebsiella from 2.6-4.5 logCFU in sinks and 1.6-4.9 logCFU/mL in drains. There was low to no correlation between occurrence of detectable bacteria in sink basin and drain water samples from the same room; median correlation estimates across ten rooms for the three units were -0.09 (-0.3; 0.46 95% CI), 0.21 (0.02; 0.67 95% CI) and 0.36 (-0.18; 0.52 95% CI) for Acinetobacter, 0.07 (-0.32; 0.45 95% CI), 0.09 (-0.32; 0.53 95% CI), and 0.18 (-0.21; 0.39 95% CI) for Pseudomonas and -0.25 (-0.3; -0.17 95% CI), 0.04 (-0.29; 0.55 95% CI), and 0.25 (-0.4; 0.54 95% CI) for Klebsiella. Conclusions Over 7 months, Acinetobacter and Pseudomonas aeruginosa were common in basins and drains of patient room sinks. Klebsiella was less common but still detected. Concentrations fluctuated over time but could reach levels up to 6-7 login the sink basin. These results suggest that sinks and drains in inpatient rooms are potentially a consistent source of HAI organisms in the hospital environment.

